# Supplementary material for: Pomegranate Peel and Olive Leaf Extracts to Optimize the Preservation of Fresh Meat: Natural Food Additives to Extend Shelf-Life
Source: Microorganisms. 2024 Jun 27;12(7):1303. doi: 10.3390/microorganisms12071303 (PMC11278528; doi:10.3390/microorganisms12071303)
Supplement: Supplementary file 1 [file microorganisms-12-01303-s001.zip › microorganisms-3077877-supplementary.pdf]

Pomegranate peel and olive leaf extracts to optimize the preservation of fresh meat: natural food additives to extend shelf-life

Giuseppina Forgione, Giuseppa Anna De Cristofaro, Daniela Sateriale, Chiara Pagliuca, Roberta Colicchio, Paola Salvatore, Marina Paolucci and Caterina Pagliarulo

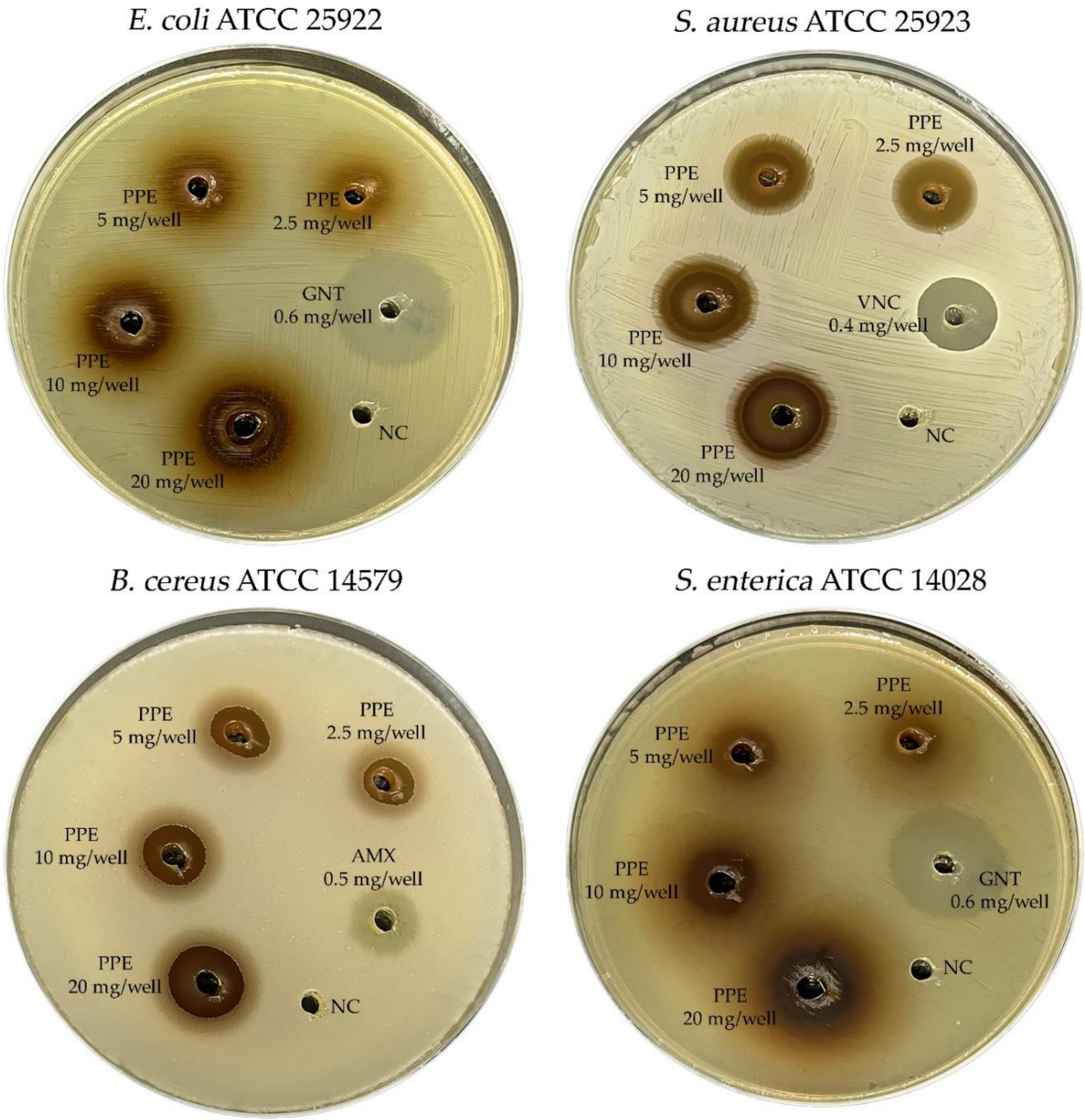

Figure S1. Images of *in vitro* antibacterial activity of PPE evaluated by the agar well diffusion method against selected microorganisms. PPE, pomegranate peel extract; NC, negative control; GNT, gentamicin; VNC, vancomycin; AMX, amoxicillin.
